# Perceiving affordances and the problem of visually indiscernible kinds

**DOI:** 10.3389/fpsyg.2024.1388852

**Published:** 2024-09-04

**Authors:** Mette Kristine Hansen

**Affiliations:** University of Bergen, Bergen, Norway

**Keywords:** affordances, perception, phenomenal consciousness, perceptual experience, high-level kinds, rich content, thin content

## Abstract

In this study, I defend the claim that we can perceptually experience what objects afford when we engage with objects belonging to natural or artificial categorical high-level kinds. Experiencing affordances perceptually positions us to act in specific ways. The main aim of this study was to argue that this view has explanatory advantages over alternative views. An increasingly popular view within the philosophy of perception, most famously defended by Susanna Siegel, claims that we sometimes visually experience natural and artificial objects as belonging to categorical high-level kinds. When visually experiencing a lemon, one does not only experience its low-level properties such as shape and color, sometimes one also experiences the object as a lemon. A challenge arises when attempting to explain what happens when one experiences an object that is experientially indistinguishable from another object, yet these objects belong to different high-level categorical kinds. For instance, if someone perceptually experiences a lemon as a lemon, her experience can be considered as accurately representing or presenting a lemon. However, if the subject perceptually experiences a lemon-shaped soap bar, which cannot be discriminated from a real lemon by sight alone, the experience is deemed inaccurate because there is no real lemon present. The problem is that such a judgment seems counterintuitive; unlike with hallucinations and illusions, there seems to be nothing wrong with how the object appears. Therefore, it is difficult to understand how the mistake could be a perceptual mistake. I will first present arguments supporting the claim that when we visually encounter objects such as lemons, we sometimes also perceive the affordances of these objects—what they provide or offer us. I will further argue that this perspective on affordances offers a more compelling explanation than other alternative accounts when it comes to our perception of visually indistinguishable objects that nonetheless belong to categorically distinct high-level kinds.

## 1 Introduction

It is widely accepted that we visually experience objects as having low-level properties such as colors, shapes, and spatial locations (Bayne, 2009; Bayne, 2011; Siegel, 2010; Siegel, 2014; Carruthers and Veillet, 2011). However, the question arises: Can we also experience objects as having other properties? Can we experience objects as belonging to various categorical high-level kinds, such as being a lemon or an apple? Or are these high-level properties inferred from our perceptual experiences of low-level properties?

In this study, I advocate for the position that our visual experience encompasses affordances, which I here understand as potentials for actions. We are capable of perceptually experiencing apples and lemons as edible, stairs, and slopes as climbable, and so forth. While I remain agnostic regarding whether we can also visually experience categorical high-level kind properties—such as lemon-hood and apple-hood—I will put forth the argument that my view circumvents several difficulties encountered by the view that we visually experience categorical high-level kinds.

First, as Nanay maintains, the view that we visually experience affordances is less vulnerable to criticism based on attentional differences (Nanay, 2011; Nanay, 2013).

Second, it can support contrast arguments employed in advocating for the view that we perceive categorical high-level kind properties, without requiring the adoption of that view.

Third, it sidesteps intellectualism, where intellectualism is characterized by the need for a relatively advanced level of conceptual competence. In this context, intellectualism should be understood as the viewpoint that performing a range of actions requires not only conceptual competences but also the ability to infer from previous beliefs. Intellectualism is a position that can be said to overcomplicate natural processes and largely disregards our evolutionary history and our kindship with animals. In addition, intellectualist positions are not compatible with several theories on the epistemology of perception. Therefore, I consider it a strength of my perspective that it can circumvent intellectualism.

Fourth, and most crucially for the arguments presented in this study, it solves the problem of indiscernible kinds—an obstacle that challenges the view that we sometimes visually experience categorical high-level kinds.

I begin with a succinct elaboration of my stance on affordances, which serves as a modified interpretation of Gibson’s view. My use of the term “affordance” differs from Gibson’s in that it is, unlike Gibson’s use, compatible with a representationalist view on conscious perception. In the next section, I marshal empirical evidence to articulate compelling reasons for the belief that we do not only perceive affordances but that they can also constitute an integral part of our visual experience.

Next, I delineate the contrast between the thin-view and the rich-view concerning the content of perceptual experience.^1^ The thin-view is advocated by philosophers such as Tye (2009), Carruthers and Veillet (2011) and Prinz (2012), while the rich-view has gained support from Siegel (2010), Bayne (2011) and Nanay (2011). I will put forth the argument that we can, at times, visually experience affordances, suggesting that our experiences are richer than what the thin-view proposes. However, aligned with the thin-view, I will argue against the notion that we perceive categorical high-level kind properties.

In the subsequent section, I delve into the theory that we perceive high-level properties, such as categorical high-level kinds (Siegel, 2010). I examine Siegel’s compelling argument from phenomenal contrast, where she posits her theory as providing the best explanation of the pronounced difference between contrasting pairs of experiences (Siegel, 2010). Nanay, however, efficaciously contends that such contrasts can be attributed to attentional variances (Nanay, 2011; Nanay, 2013). Notwithstanding, Siegel might counterargue that her explanation for the contrast remains superior.

After reviewing Nanay’s points, I address a more formidable challenge to the view that asserts that we can perceptually experience categorical high-level kinds: the problem of visually indistinguishable kinds. I will argue that while this poses a significant challenge to the view that we can perceive categorical high-level kinds, it does not trouble the affordance view I endorse. My perspective not only sidesteps the indistinguishability problem but also steers clear of intellectualism and exhibits resilience against critique based on attentional differences.

## 2 Affordances

Traditionally, perception has been considered as a passive process, where the perceiver is merely an observer of a scene with no need to interact (see, e.g., Marr, 1976; Marr, 1982). This passive view contrasts with an active perspective on perception, according to which the perceiver engages with her environment by discerning ways to relate to and act upon things in the world. Gibson, well-known for introducing the concept of affordances, argued that when we engage with the world through perception, we perceive the possibilities our immediate environment and its objects offer for action (Gibson, 1979). We are not passively absorbing information about the scenery; rather, we see ways of relating to and acting upon things in our environment:

The affordances of the environment are what it offers the animal, what it provides or furnishes, either for good or ill. The verb to afford is found in the dictionary, but the noun affordance is not. I have made it up. I mean by it something that refers to both the environment and the animal in a way that no existing term does. It implies the complementarity of the animal and the environment (Gibson, 1979, p. 127).

For Gibson, the affordances of the environment are offerings and threats that the environment solicits to animals who are living in that environment, relative to the abilities of the animal. For instance, an apple is edible, a rock is climbable, a hole in the ground offers hiding, and a cliff offers the threat of being fall-of-able. While I concur with Gibson on the perceptibility of affordances, I distance myself from certain elements of his theory of perception (See also Nanay, 2011; Nanay, 2013; McClelland, 2019; Vetter, 2020). First, Gibson held that we do not perceive objects, but we perceive affordances, whereas I maintain that we indeed perceive objects and, in addition, what these objects afford. Second, Gibson imbued affordances with normative implications, suggesting that the property of affording an action A is indicative of what we should or ought to do. My stance is more nuanced. I contend that when we perceive affordances, we do not perceive prescriptive actions but rather possibilities, such as perceiving an apple as edible signifies the potential to eat it, not an obligation to do so. Third, Gibson claimed that “a postbox affords letter-mailing to letter-writing humans in a community with a postal system” (Gibson, 1979, p. 139). This could, however, be considered an over-intellectualization of the nature of perception, wherein such understanding does not derive from perception itself but is inferred from it (Prosser, 2011; Nanay, 2011; Nanay, 2013).

Although my perspective remains flexible on the range of affordances we perceive, this study will concentrate on simpler and more universally recognized cases. I propose that we can perceive apples, lemons, and oranges as edible, stairs as climbable, chairs as sittable, and similar straightforward cases. This does not imply, however, that I believe we can perceive complex cases such as wars as winnable or examinations as solvable through perception alone.

Regarding the nature of affordances, I take them to be relational properties, yet I refrain from adopting a definitive stance on their precise ontological status (for a deeper exploration, see Vetter, 2020). Affordances are considered relational because they are properties of external objects and environments that are perceivable by creatures with certain abilities and/or dispositions. The perception of a hill as climbable, for example, depends not only on the characteristics of the hill but also on the capacity of the perceiver. In my view, individuals can experience objects or spaces in the environment as action-enabling. An individual, subject S, at a given time t, and within certain conditions C, may experience an object or environmental feature as action-enabling/A-able if it is not impossible for S to execute action A at time t in C (See Nanay, 2011; Nanay, 2013 for further elaboration).

Contrary to popular views at the time, Gibson held that affordances are directly perceived. His view is in line with direct realism and, to an even greater extent, with an embodied approach to perception (Noë, 2004). In a divergence from Gibson’s view, I maintain that my conceptualization of affordances can also be reconciled with the representationalist approach to perception. Representationalists claim that perceptual states possess contents framed by conditions of accuracy. Throughout this study, while I remain agnostic on the debate between direct realism, and embodied theories of perception and representationalism, I will employ representationalist language, suggesting that the content of perception may encompass action-related properties or affordances (See also Nanay, 2011; Nanay, 2013).^2^ Nevertheless, the arguments presented here could be reformulated to align with the frameworks of both direct realism and embodied theories of perception.

We can perceive apples as edible and slopes as climbable; however, it is not necessary for every affordance to be immediately detectable. Affordances are perceived insofar as they constitute an integral part of our perceptual experience—making a constitutive, rather than merely a causal, contribution to our experience. Adhering to the representationalist framework, perception is understood to have contents that represent states of affairs. My approach suggests that when we perceive an object, we can be directly aware of the potential interactions it affords based on our capabilities and the object’s properties. This perceptual content, which includes affordances, is shaped by the nature of our engagement with our environment.

Perceptual experiences are phenomenally conscious perceptual states in which there is something that it is like for the subject of the experience to be in that state. I refer to this as the perceptual states’ phenomenal character (Hansen, 2018). While not all perception is phenomenally conscious, this study specifically addresses perceptual experience. It is acknowledged that affordance perception can occur subliminally in some, or perhaps many, instances. Nonetheless, these instances fall outside the scope of this discussion. My focus here is on our perceptual experience, and I propose that perceiving affordances can sometimes contribute to the phenomenal character of our perceptual experiences.

To say that a perceptual experience has content is to assert that it has specific conditions of accuracy, with these conditions stipulating when the experience can be deemed veridical (Siegel, 2021). The accuracy or inaccuracy of the perceptual experience co-varies with its correspondence to the actual states of affairs—the veridicality or fallaciousness of the experience. The accuracy conditions determine when, and under which circumstances, the experience is veridical. For instance, when a subject perceives that ‘there is an orange cat on the mat’, the experience is considered veridical if, in fact, an orange cat is present on the mat. Conversely, the experience is deemed non-veridical if this is not the case.

In the following section, I will provide several arguments to support the notion that we do perceive affordances and, on occasion, consciously experience them.

## 3 Perceiving affordances

An expanding body of empirical research bolsters the notion that we perceive affordances (with studies by Schindler et al., 2004; Himmelbach and Karnath, 2005; Young, 2006; Ye et al., 2009; Mark et al., 2015; Wagman et al., 2016; Gadsby, 2017; Seifert et al., 2021). Under specific conditions, the mere sight of a football may activate the motor processes involved in kicking the ball, regardless of any deliberate intentions on the part of the perceiving agent to do so. Perception of action possibilities prompts the initiation of motor processes required for executing the action. There are compelling reasons to support the idea that affordances can be perceived and not inferred by the perceiving subject (McClelland, 2019). Yet, it remains an open question: Do affordances play a role in our conscious visual experiences, or are they merely processed at a perceptual level without reaching our conscious awareness?

In this section, I will present arguments and empirical evidence to substantiate the claim that we not only perceive affordances but also encompass a part of our perceptual experience. While some may contend that this evidence is not conclusive, I will maintain that the affordance perspective offers a straightforward and coherent explanation for the empirical observations. Considering that the affordance view provides a sound explanation of the empirical observations, the burden of proof now falls on the rival views to demonstrate their capability to achieve the same.

Evidence suggests that humans, along with other animals, possess two functionally distinct yet complementary visual pathways (Milner and Goodale, 1995). The ventral stream is related to the inferotemporal cortex, whereas the dorsal stream relates to the posterior parietal cortex. Miller and Goodale propose that the ventral stream is involved in conscious perception and recognition of objects, while the dorsal stream is thought to supply information for the visual guidance of action. While playing a vital role in visuomotor actions and the subject’s adaption to the environment, there is some indication that information processed along the dorsal stream may have restricted access to consciousness (Milner and Goodale, 1995). Conversely, the ventral stream plays a crucial role in consciously recognizing objects, influencing decisions based on actions that showcase an understanding of an object’s function, for instance, grasping a hammer by the handle rather than the head (Young, 2006). However, as argued by Carruthers, the two distinct systems are not merely designed to process complementary streams of information related to visual stimulus perception (Carruthers, 2000). Instead, each system serves two distinct modes of operation, which, when combined actively, contribute to our daily interaction with the world.

In his attempt to reconcile Gibson’s view on perception with the traditional observer view (Marr, 1982), Norman claims that affordances are processed by the dorsal stream (Norman, 2002). The dorsal stream functioning does not involve the processing of representations. Should affordances be linked with the non-conscious processing characteristic of the dorsal stream, this could bolster the argument that the perception of affordances might operate outside our conscious awareness (Norman, 2002). Various empirical studies seem to provide backing for this claim:

“When we reach out for an object, for example to pick up a cup, we use a set of exquisitely calibrated visuomotor processes in our brains that unthinkingly take into account the location and physical properties of the tar-get object as well as the location and state of the body, arm and hand. Neurophysiological and functional MRI studies show that these brain systems are largely located around the so called ‘dorsal stream’” (Schindler et al., 2004, p. 779).

Nevertheless, as Young contends, a significant amount of evidence derived from case studies on visual pathology indicates that only specific affordances are processed via the dorsal stream (Young, 2006). Affordance processing also engages the ventral stream. Young proposes that affordances can be categorized into two types: those that guide the selection of an action and those that govern the execution of the action itself.

Patient DF, who was studied by Milner and Goodale, suffers from visual agnosia due to carbon monoxide poisoning. She lacks the ability to see objects, instead perceiving only a muddled blend of colors and textures. Despite her incapability to recognize objects, she remains capable of engaging in visuomotor tasks such as grasping. Remarkably, DF could reliably insert a letter through a mail slot, even though she was unable to report the slot’s location when questioned by the examiner (Goodale et al., 1991). In a separate study, DF was tasked with estimating the sizes of various objects. Her performance on this task did not exceed random guessing. However, DF demonstrated high proficiency in real-time actions such as grasping and reaching for these objects. Milner and Goodale hypothesized that DF’s agnosia might be attributed to damage in the ventral stream of her visual system while her intact dorsal stream could account for her ability to execute actions such as grasping and reaching (Milner and Goodale, 1995). This hypothesis gained further support from MRI scans of DF’s brain which provided confirming evidence (James et al., 2003). Arguably, DF can pick out affordances from within the optic array due to the functioning of her dorsal perceptual stream. However, while DF demonstrated proficiency in grasping objects regardless of form, shape, or location, she exhibited a deficiency in selecting the optimal part of the object for effective use. Appropriate grasping of, for example, a hammer requires that one grasps it by the handle. DF was unable to be guided by knowledge of the function of the object when grasping it. Drawing on these findings, Cary et al. claim that DF does not utilize affordances during the act of grasping because she does not possess knowledge about the function of the objects (Carey et al., 1996). However, Young’s distinction between different types of affordances offers a more credible interpretation of her capabilities (Young, 2006). This perspective suggests that DF is able to discern affordances related to the immediate physical action of grasping and reaching for objects, yet she is incapable of perceiving affordances that pertain to an object’s function. While certain affordances may be processed subconsciously, this is not the case for all affordance types. The perception of some affordances necessitates the operation of both the ventral stream and the dorsal steam within the visual system.

Another patient IG, studied by Milner et al., contrasts with DF as she is unable to locate and grasp an object appropriately, even though she can identify the use and function of the object (Milner et al., 2001). Though she struggles with real-time grasping, she excels in performing pantomimed actions. Milner et al. conclude that IG’s pantomime ability constitutes evidence for some form of ‘off-line’ visuomotor guidance operating independently of the dorsal stream projection to the posterior parietal cortex. Both Westwood and Goodale, as well as Himmelbach and Karnath, claim that the evidence from studies of patients suggests that there is an interaction between the two visual systems (Westwood and Goodale, 2003; Himmelbach and Karnath, 2005). According to Young’s theory, it appears that various affordances are not confined to a single neurological pathway within the visual system. Some affordances, processed by the dorsal system, are perceived without conscious awareness, whereas others, relying on knowledge of the object’s function, engage the ventral stream. Hence, affordances that are processed along the dorsal stream are registered subconsciously. In contrast, those processed through the ventral stream contribute to the subject’s phenomenal experience (Young, 2006).

Furthermore, recent studies have reinforced Young’s perspective, challenging the previously held belief in strictly separate visual systems by demonstrating dynamic interactions between them (De Haan et al., 2013, 2017; Goodale and Milner, 2018; Ferretti, 2019, 2020, 2021). For instance, the research conducted by Ye et al. suggests that an individual can discern which action mode is viable among various affordances, as well as how to modulate their movement to accomplish the intended task goal (Ye et al., 2009). Echoing Ferretti, it is the ventral stream’s task to orchestrate action planning which involves selecting targets for action. Concurrently, the dorsal stream’s role in motor programming is to calculate the parameters necessary for effectively guiding actions toward those targets (Ferretti, 2021).

As demonstrated, there is empirical evidence to support the notion that we perceive affordances and that the pertinent perceptual processing incorporates not just the dorsal stream but also the involvement of the ventral stream. Moreover, Nanay provides additional persuasive reasons to believe that affordances can be part of our perceptual experience. Drawing from studies on patients with unilateral neglect, Nanay offers evidence suggesting that these subjects can experience affordances (Nanay, 2011). Patients are either slow or unable to locate objects defined by their salient visual properties such as colors and shapes. Yet they are capable of, and efficient at, locating objects defined by the action for which the object can be used. Their experiences of what objects afford cannot be inferred from experiences of low-level properties such as shapes and colors since they do not have experiences of such properties.

The view that we perceive affordances also fits perceptual phenomenology, that is, what our experiences are like (McClelland, 2019; Vetter, 2020). Looking at the cup in front of me, I seem to perceive that the cup affords grabbing. If in danger, the hole in the ground that affords hiding seems more salient than the colors and shapes of the ground. It is likely that evolution has shaped perception so that it enhances fitness and thus our ability to survive. Affordances are action possibilities that animals need to become aware of to survive and thrive (Chemero, 2003; Wagman et al., 2016).

## 4 Inference or experience: thin- versus rich-views

Following the traditional thin-view on perception, affordances are not perceivable by a human or an animal: “…perception of affordance is infused or meditated from perception of *low-level properties* such as shape, color, etc.” (Fodor and Pylyshyn, 1981, p. 145). According to this perspective, affordances are not strictly perceived but are instead deduced from perceptual cues such as colors, shapes, and motions. This conventional view stands in contrast to Gibson’s theory, which posits that perceiving is an interactive process involving actions and movements (Gibson, 1979; Chemero, 2003; Noë, 2004; Prosser, 2011; Nanay, 2011; Nanay, 2013; Siegel, 2014; McClelland, 2019; Vetter, 2020). From this perspective, our perceptual experience extends beyond the simple detection of shapes and colors; it encompasses the apprehension of information about our environment, including opportunities for action, or affordances, it presents to us.

Advocates of the thin-view on perceptual experiences contend that the contents of such experiences are limited to low-level properties, such as colors, texture, spatial, temporal properties, motion, and the like (See Tye, 2000; Tye, 2009; Carruthers and Veillet, 2011; Prinz, 2012; Brogaard, 2013; Hansen, 2018). In contrast to the thin-view, the rich-view maintains that the content of our experiences can include more complex high-level features as well, such as personal identity, natural and artificial kind properties, semantic properties, and causation (see Siegel, 2010; Siegel, 2014; Bayne, 2009; McClelland, 2016; Nes, 2016; Hansen, 2018). The thin-view asserts that the phenomenal character of visual experience is limited to representing low-level properties. In contrast, the rich-view argues that the phenomenal character can encompass high-level properties, such as high-level natural and artificial kinds, and this is part and parcel of perception’s phenomenal character (Bayne, 2009). The thin-view posits that the recognition of high-level categorical kinds, such as identifying an object as a lemon, occurs at the cognitive level rather than within the immediate scope of perceptual experience (Fodor and Pylyshyn, 1981). Dretske made a notable distinction between ‘seeing things’—which is associated with perceptual experiences—and ‘seeing facts’—which is associated with perceptual beliefs (Dredske, 1993). Dretske claims that it is possible to perceptually experience an armadillo crossing the road without consciously acknowledging the fact that an armadillo is crossing the road (Dredske, 1993). We may experience the event of the armadillo crossing the road, and from this experience, we come to judge or infer that an armadillo is crossing the road—if we possess the concept of armadillo. The experience itself is a component of perception, whereas the act of making the judgment is cognitive, rather than perceptual, in nature.

The identification of the animal crossing the road as an armadillo is derived from an inference that combines our perception of low-level properties—such as shape, colors, movement, and texture—with our existing knowledge and conceptual capacity. Nevertheless, proponents of the thin-view acknowledge that high-level kind recognition may still have an indirect impact on our perceptual experiences. This influence can manifest through mechanisms such as attention or subconscious top–down processing, which can modify the way we attend to and interpret the sensory information we receive (Prinz, 2012).

Regarding some properties, it is challenging to draw a definitive line between low-level properties and high-level properties. Consequently, when differentiating between the rich-view and the thin-view, it is common to refer to examples of properties that are unmistakably high-level, such as personal identity and natural kind properties, and those that are indisputably low-level, such as colors and shapes. Affordances and gestalt properties represent the attributes that might be considered as borderline, as they are not readily classified as either distinctly high-level or purely low-level, but rather inhabit a gray area in between.^3^

While experiencing properties such as high-level kinds requires that the experiencing subject has a recognitional concept referring to the kind, such a requirement is not needed for experiencing affordances. Carruthers and Veillet convincingly propose that if it is the case that we experience high-level kind properties, the content of perceptual experience must be conceptual in nature (See Carruthers and Veillet, 2011). However, by contrast, the idea that we perceive affordances—that is the action possibilities an environment presents—does not require such a conceptual foundation. The perception of affordances is thus compatible with the notion that the contents of our perceptual experiences can be non-conceptual, just as it can align with the conceptual view.^4^

One advantage of the thin-view over the rich-view is the former’s compatibility with a broader range of theories concerning the content of perception. It is also inherently anti-intellectualist. However, as I will demonstrate, the view that we perceive basic affordances preserves all these strengths while also offering the explanatory benefits associated with the rich-view.

## 5 The argument from phenomenal contrast

The rich-view posits that we *sometimes* experience high-level properties such as natural and artificial categorical high-level kind properties when we have experiences of things that belong to such kinds (see Siegel, 2010). Conversely, the thin-view maintains that we never experience such properties. While there are claims that introspection may help us adjudicate between these views, the reliability of introspection as a discerning tool in this context is debatable (Schwitzgebel, 2019). Advocates of the thin-view report that upon introspecting their perceptual experiences, they do not detect high-level properties. On the other hand, supporters of the rich-view assert the exact contrary—that they do experience such properties. Given the conflicting nature of these introspective intuitions, striving for a resolution requires seeking out arguments that extend beyond mere introspection to avoid an impasse.

Contrast arguments are arguments from the best explanation. I take it that the best explanation will be the claim that on balance does best in respect to virtues such as simplicity, generality, being more advanced in terms of explanation, and coherence with well-established theories (Hansen, 2018, p. 304). While contrast arguments are widely used to support various claims about the nature of conscious experiences, the relevant contrast arguments here are those used to argue for the claim that we can have perceptual experiences involving different categorical high-level kinds. The first step when forming a contrast argument is to clarify what, for instance, what can be explained by the claim that we experience high-level kind properties. The second step is to see whether this claim provides us with the best explanation, compared with alternative explanations. This method gives us a way of limiting the use of introspection, though our conclusion will still partly depend on introspective intuition. To form a contrast argument, we need to find a pair of contrasting experiences with the following features (Hansen, 2018, pp. 304–305):

1. The experiences must be minimal pairs: That is, they are experiences that are of the same object belonging to a categorical high-level kind, under the same viewing conditions, where nevertheless, there is a slight but noticeable phenomenal difference between the experiences (Koksvik, 2015).
2. The overall experience in one of the pair seems to include a representation of a kind property, such as ‘lemon,’ while the other does not.
3. The phenomenal characters of the two overall experiences are evidently distinct, compelling one to acknowledge the presence of a phenomenal contrast between them, regardless of any initial disagreement toward the proposed explanans of this contrast.

If the claim under examination provides the most compelling explanation for the phenomenal contrast, then—following the principle of the best explanation—one of the experiences will involve a categorical high-level kind, while the other will not. This follows because there will be a disparity in the phenomenal characters of the two experiences that, based on the best explanation, can be attributed to the representation of categorical high-level kind properties in one of the experiences but not in the other.

An obvious strategy to refute a particular contrast argument is to argue that there is no phenomenal contrast between the two experiences. Whether such a strategy is successful or not depends on the cases one is considering. Even if one agrees that contrast exists in the cases one is presented with, there are at least two different approaches to counteract a strong claim of phenomenal contrast (Hansen, 2018):

1. Claim that the phenomenal contrast is not a contrast at the level of visual experience but rather a contrast in non-visual phenomenology (see Montague, 2017; Montague, 2023).
2. Admit that there is a contrast but deny that this contrast is due to experience of categorical high-level kind properties.

In the next sections, I will mainly focus on the second option. I have already argued in § 3.0 that we can experience affordances. When it comes to the cases Siegel appeals to when arguing that we can visually experience categorical high-level kinds, I choose to assume without appreciation that the phenomenal contrast is visual. First, based on phenomenology, if there is a contrast in these cases, then phenomenally the contrast seems to be visual. Second, alternative explanations for the contrasts in the cases are more contentious than Siegel’s view. Montague argues that the contrast is a contrast in cognitive phenomenology (Montague, 2017; Montague, 2023), yet the very existence of cognitive phenomenology is debatable. Third, it is necessary to constrain the scope of this study.

Therefore, in the forthcoming sections, I will explore the second option. I will argue that the phenomenal contrast observed in the proposed contrasting cases can be accounted for by the perceptual experience of affordances rather than the perception of categorical high-level kinds.

### 5.1 The expert-novice argument

Susanna Siegel famously appeals to expert-novice cases when presenting contrast arguments in support of her position:

“Suppose you have never seen a pine tree before, and are hired to cut down all the pine trees in a grove containing trees of many sorts. Someone points out to you which trees are pine trees. Some weeks pass, and your disposition to distinguish pine trees from the other improve. Eventually, you can spot the pine trees immediately. They become salient to you…Gaining this recognitional disposition is reflected in a phenomenological difference between the visual experiences you had before and after the recognitional disposition was fully developed” (Siegel, 2006, p. 491).

We can label the novice’s experience as ‘e’ and the expert’s experience—as one becomes an expert—as ‘e*’. Following Siegel’s argument, there is a phenomenal contrast between these experiences, and this contrast is due to the expert’s ability to experientially pick out the kind ‘pine tree’.

Nevertheless, even if we concur with Siegel that the expert’s and novice’s experiences differ in phenomenal character (and that the difference is visual in nature), it is not necessarily the case that this difference stems from the expert’s experience of categorical high-level kinds. One may alternatively claim that when becoming an expert one learns to attend to the properties of the pine tree in a different way. For instance, one learns to pick out those shapes, colors, and textures that are characteristic of pine trees (Hawley and Macpherson, 2011; Nanay, 2011; Nanay, 2013; Hansen, 2018). Thus, the difference in the experiences between the expert and novice can be explained as a variance in the low-level properties one is able to visually discern through attention. This discrepancy in attentional capabilities does not necessarily have to be within one’s conscious awareness. After acquiring the concept of ‘pine tree’ by learning to differentiate pines from other trees, the act of identifying these trees gradually becomes habitual and thus automated. One is not consciously aware of the changes in how one attends to the trees and their properties.

It is worth noting that we can assume the viewing conditions are the same in experiences e and e*. Thus, there is no difference in aspects such as lighting conditions or the visual capabilities of the person when experiencing the pine tree as a novice versus as an expert. However, this will not negate the claim that the difference in phenomenal character is due to the expert’s ability to attend to low-level properties that the novice fails to notice. To assert that attention is also constant would be begging the question and is unlikely, based on our understanding of how attention functions (Nanay, 2011). What we need to consider when evaluating these cases is whether this appears to be the *best* explanation for the contrast and whether it can *fully* account for it.

Attention clearly plays a significant role in many expert/novice cases. For instance, during pregnancy, one usually visits the hospital for several ultrasound examinations to ensure that the fetus is developing properly. While looking at the images, the doctor or midwife, who has extensive experience and expertise in interpreting them, can discern features represented there that you, as a novice, are unable to recognize, even though you are examining the same picture. However, the doctor can guide you and point out the relevant features, enabling you to, for example, identify the sex of the fetus and similar details. When guided by the expert in this way, it seems intuitive that the difference between our experience and that of the expert becomes less.

Is it still likely that the best explanation for the contrast in these cases is due to more than just differences in attentional abilities? Does the expert experience the image differently from the novice, even when the expert points out the relevant properties to which the novice should attend? And if so, is the difference best explained as a variance in the experiences of high-level kinds?

The rich-view appears to hold certain advantages over the thin-view as the former appears to better align with introspective insights. According to the thin-view, based on her experiences, an expert is able to more easily infer and judge that the tree she is seeing is a pine, compared to a novice. However, when observing objects belonging to various familiar categorical high-level kinds, it does not feel like one is making any inferences. Based on introspection, it feels like the experience is direct, in such a way that no inferences are necessary. Nonetheless, advocates of the thin-view may argue that the inference occurs very rapidly and unconsciously; thus, the subject is not aware of making such an inference. However, the rich-view seems to offer a simpler and more elegant explanation of what occurs in expert cases compared to the thin-view, and as previously mentioned, it is better aligned with introspection. Consequently, the rich-view arguably outperforms the thin-view in accommodating contrast arguments.

However, in the following section, I will point out a challenge that confronts rich-views that assert that we can experience categorical high-level kinds, namely, the problem of the indiscernibility of kinds. I will further discuss how proponents of the rich-view have attempted to address this issue by appealing to Fregean content, and I will demonstrate why these attempts fail to resolve the problem. As a constructive contribution to this debate, I will advocate for the affordance view, which effectively accounts for the phenomenal differences between the experts’ and the novices’ experiences and adeptly handles the problem of indiscernible kinds. I will conclude that the affordance view has greater explanatory potential than both the rich-view and the thin-view.

## 6 The problem of visually indiscernible kinds

*“In the night, imagining some fear, How easy is a bush supposed as a bear!”* (Shakespeare, W. A Midsummer Night’s Dream, V. I) (See Shakespeare, 2024).

If we accept the view that we sometimes visually experience categorical kind properties, there can be a phenomenal difference between experiencing a ‘lemon’ as a lemon and merely experiencing the lemon without recognizing it as such. Similarly, there can be a difference in experiencing a glass of vodka as ‘a glass of vodka’ and experiencing a glass of water as ‘a glass of water’.

However, when visually presented with a glass containing either vodka or water and asked what it is, I can only make a guess (Hansen, 2018). A glass of water may be *visually* indistinguishable from a glass of vodka, even to a vodka expert. When I am at a Russian bar, and I visually experience what is in fact a glass of water as a glass of vodka, am I visually misrepresenting the transparent liquid in the glass?

The Shakespearean quote above acknowledges that we often mistake one object for another due to problematic viewing conditions and emotional influences. However, when I mistake a glass of water for a glass of vodka based on my visual experience, my error is not a result of problematic viewing conditions or emotions. I can make the mistake regardless of such factors.

An advocate of the rich-view on categorical high-level kind experience might adopt an internalist stance on intentional content and argue that when I view an object as belonging to a certain categorical high-level kind, I am disposed to apply a corresponding concept to that object. Although the external information contributing to my experiences of vodka and water is the same, the specific concept that I am disposed to apply to this information plays a constitutive role in shaping the phenomenal character of my experience. Consequently, my experience is deemed inaccurate if I erroneously experience a glass of water as a glass of vodka. Nevertheless, it seems that the reason glasses of water and glasses of vodka are visually indiscernible is that they share the same visually detectable properties. This issue is further discussed in the forthcoming section. If I am visually presented with two glasses containing transparent liquids and am later informed that the glass to the right contains water and the glass to the left contains vodka, regardless of this new information, the two glasses continue to *look* indiscernible to me. The case of different categorical kinds that are nevertheless visually indiscernible seems to provide a compelling argument for the thin-view: It appears that my visual experience is not inaccurate when I ‘experience’ the glass of water as a glass of vodka. Instead, the error in kind identification occurs at the level of belief; I am mistaken when I believe that the glass I am seeing is a glass of vodka. Could the advocate of the rich-view, based on her claim that we *sometimes* experience categorical high-level kind properties, assert that the case of seeing glasses of vodka or water does not entail experiences of categorical high-level kinds? Or, alternatively, might she argue that we do experience categorical high-level kinds in these cases, but the categorical high-level kind we visually experience when seeing glasses of vodka and glasses of water is a shared category, such as ‘transparent liquid’?

The challenge for the proponent of the rich-view is that we can envision indiscernibility scenarios for nearly any object belonging to a categorical high-level kind. There are lemon-shaped soap bars that look (at least under certain viewing conditions) exactly like real lemons. There are plastic apples used for decoration that look like real apples and there are plastic sushi pieces that can be mistaken for real sushi, to name just a few examples.

Even if the proponent of the rich-view asserts that we only *sometimes* experience categorical high-level kinds, it may be difficult to determine when we do and when we do not. It might appear that our error in identifying visually indiscernible objects belonging to different kinds is not a perceptual mistake. Unlike cases of illusion, such as that portrayed in “A Midsummer Night’s Dream,” misidentification can occur regardless of factors such as viewing conditions and emotions.

### 6.1 Solving the problem by appealing to Fregean content?

According to Bayne, the indiscernibility problem need not be a concern for those who subscribe to the Fregean-content view, while it remains a problem for those who adopt a Russellian view of the content of perceptual experiences (Bayne, 2011). To address Bayne’s argument effectively, we need to clarify what distinguishes these views.

The strongest version of the Russellian content view posits that the content of perceptual experience compromises the object that appears to one as possessing certain properties and the properties that it appears to possess (Siegel, 2021). For example, if one sees a green circle, the content of one’s experience will be the structured proposition containing the object that appears to be a green circle and the property of being a green circle. This content can be expressed as [o, P], where ‘o’ is the object, and ‘P’ is the property that ‘o’ seems to have. Such a Russellian view involves both objects and properties. However, most philosophers who think that the content of perceptual experience is Russellian hold a weaker Russellian view where the content is said to be existentially quantified content. That is, the content can be expressed as ‘there is a green circle there’.^5^

The Fregean view holds that the content of perceptual experiences is composed of *modes of presentations* of objects and properties, rather than the objects and properties themselves (Chalmers, 2004). This view has the virtue of being able to explain the differences in experiences of two people who experience and represent the same green circle at the same location but from different perspectives (Schellenberg, 2018). According to the Fregean perspective, these experiences contain different modes of presentation of the circle (Siegel, 2021). The Fregean view can also explain perceptual constancies. The table in my living room appears uniformly white, even though I can see variations of lightness and darkness when I look at it. A Fregean might claim that my experience represents the whiteness of the table under modes of presentation that may vary with variations of light and shadow (Siegel, 2021). Now, following Bayne’s example, a Russellian will claim that a non-tiger that looks like a tiger must be misrepresented if tiger-hood is something we can visually experience, while the Fregean can avoid this counterintuitive result (Bayne, 2011, p. 29). The idea seems to be that in the case of seeing the visually indiscernible non-tiger and mistaking it for a tiger, the non-tiger is presented under a mode of presentation that both tigers and tiger-lookalikes can be presented under.

However, one concern with Bayne’s proposal is that it is already quite unclear what modes of presentations are, that is, it is challenging to specify precisely what they are in each case of experiencing (see Chalmers, 2004; Prosser, 2011 for suggested explanations). The examples that are commonly cited as modes of presentation, such as seeing green circles from different perspectives and seeing the table as uniformly white, do not clarify what the common mode of presentation would be for tigers and tiger-lookalikes, or for lemons and lemon-shaped soap bars. When asked why they look indistinguishable, it is natural to attribute this to their sharing the same low-level properties. Regarding the table appearing uniformly white, one reason we might experience it as such is based on earlier experiences of interacting with the object or similar objects, where our engagement involves viewing it from different angles. Is the lemon-shaped soap bar presented to us under a lemon-like mode of presentation? Does this mean that my experience involves the concept of ‘lemon’, and that this concept is applied to the thing I am experiencing?

The Fregean attempt to avoid the problem of indiscernibility seems to lead to an acceptance of the view that experiential content is conceptual, at least in some cases. As Carruthers and Veillet argue, if one accepts the rich-view, one must also at least accept that concepts make a constitutive contribution to the phenomenal character of our experiences (Carruthers and Veillet, 2011). Lemons and lemon-lookalikes and tigers and tiger-lookalikes share all visually detectable low-level properties, so what distinguishes them seems to be the concept that one correctly or incorrectly applies to them. However, this is not in itself a reason for rejecting the view; it makes the view less attractive as it is incompatible with the non-conceptualism of experiential content. Furthermore, as it is already challenging for the Fregean to explain what modes of presentations are, extending the notion to include more than such things as constancy and location does not make this task less challenging.

A possible suggestion for a Fregean is to instead accept that we experience affordances: thus, when experiencing the lemon soap bar, one is experiencing it as being edible (or used for the same purposes as real lemons). The mode of presentation common to lemons and lemon-lookalikes is their perceived use. In the next section, I will present further arguments for the view that the contrasting cases appealed to when arguing for the experiential representation of categorical high-level kinds should instead be explained by appealing to affordance experiences. The affordance view can encompass both the contrast arguments described in § 5 and avoid the problem of indiscernible kinds.

### 6.2 Perceiving affordances in contrast cases

The argument from the indiscernibility of categorical high-level kinds seems to favor the thin-view. However, it is at least unclear whether the thin-view provides the best explanation of the phenomenal contrast between expert and novice experiences. Arguably, it does not. An expert can describe and point out to the novice what she should attend to. Still, as several studies suggest, appealing to differences in attention is not sufficient to explain the difference between expert and novice perception (See West et al., 2010; Reppa et al., 2012; Seifert et al., 2021).

As I have argued earlier, there are reasons to believe that we *sometimes* not only perceive but also experience affordances. By ‘experiencing affordances’, I mean that perceiving them makes a constitutive contribution to the phenomenal character of our visual experience. The view that we experience affordances has the advantage of being able to plausibly account for both expert/novice experiences and experiences of indiscernible kinds.

In expert-novice cases, there is a difference in what actions the expert and the novice experience the objects as affording. For instance, when the expert sees a pine tree, she experiences it as affording cutting, while the novice may not experience the pine tree as affording such actions. In the case of seeing an apple, one experiences it as being edible, to be used when making food (See also Nanay, 2011). There exists substantial evidence supporting the notion that there is a discernible difference in affordance perception between experts and novices (See, e.g., Weast et al., 2011; Reppa et al., 2012; Lehrig et al., 2019; Seifert et al., 2021). Studies of novice and expert athletes indicate differences in both motor responses and action planning. Pezzulo et al., studying differences in the performance of expert and novice climbers, conclude that:

“The better recall of Experts compared to Novices is totally due to the fact that, given that they were able to climb the difficult route, they could mentally simulate climbing (do the ‘affordances calculus’) and, with the help of the affordances, they were able to recall the sequence of required movements. Novices were impeded from simulating because they did not possess the motor capability to climb the Difficult Route. This suggests that the ability to simulate is modulated by previous motor experiences, in keeping with ideomotor theories of perception and action” (Pezzulo et al., 2010, p. 12).

Differences in perception and experiences of affordances can very well explain the contrast appealed to in contrast cases. Moreover, as evidence suggests, we have reasons to believe that such a difference will occur in such cases, in addition to attentional differences in one’s focus on the relevant object.

When mistaking a lemon-shaped soap bar for a real lemon, the mistake can be explained as an error in perceiving the actions that the object affords. This perspective seems to offer a better explanation of these cases compared to the view that the error is a mistaken identification at the perceptual level of the high-level kind to which the object belongs. In normal circumstances, or the circumstances we are accustomed to, things that look like lemons are lemons and afford eating. In the circumstances typical for evolutionary selection, a ‘look-alike’ hole in the ground affords hiding for the animal that visually experiences it as necessary for concealment (Chemero, 2003). When knowing that the glass to my left is a glass of water while the glass to my right is a glass of vodka, the two glasses may still appear indiscernible in respect to their low-level properties. However, I can experience them as affording different actions. The same holds true when I am made aware that the lemon-shaped object is really a soap bar. The soap bar affords being used for cleaning my hands, unlike the lemon, which affords being eaten or used in cooking. When experiencing tigers or tiger-look-alikes, both scenarios may afford hiding and positioning oneself at a safe distance from the animal.

By accepting that we can visually experience affordances, we can very well account for contrasts appealed to in famous contrast cases that favor the rich-view about experiences of categorical high-level kinds. As Nanay convincingly argues, contrast arguments appealing to affordances are less vulnerable from replies that point out attentional differences (Nanay, 2011). There is a phenomenal difference between seeing a tree as climbable for me versus seeing the tree as climbable for you. Nanay further shows that these differences must be experiential differences, and not ones inferred from experiences of low-level properties (such as shapes and colors), since they can be experienced by people suffering from unilateral neglect who are unable to experience such low-level properties (Nanay, 2011). It is implausible that patients with unilateral neglect can have such experiences directly, while the experiences of people with normal visions are based on inferences.

A further advantage of the affordance view over the rich-view is that it can maintain that the content of perceptual experience is non-conceptual. In addition, the affordance view is compatible with externalist views on the content of perception, as well as with direct realism and embodiment theories. It is also compatible with the rich-content view as it is perfectly possible to accept both views (see Siegel, 2014). The rich-view seems necessarily linked to intentionalism, as it is hard to see how one can claim that properties such as being a lemon can be perceived directly, since one needs to have a concept of lemon to perceive the object as such. I conclude that the view that we sometimes experience affordances has explanatory advantages over both the rich-content view and the thin-content view.

A major function of perception is to guide action. Our environment appears to us in a certain way to guide our actions appropriately. There is no need to determine whether a cup can be grabbed, or a stair can be climbed before performing these actions (McClelland, 2019). When perceiving danger, animals do not need to figure out whether a hole in the ground will function as a hiding place (Chemero, 2003). Perception can guide our actions with little or no help from conscious cognition. Affordances such as being grabbable, climbable, and hidable are perceivable by the subject. We do not merely perceive shapes, colors, and locations and infer from these properties that the cup is grabbable, the stair is climbable, and the hole affords hiding (Clark, 2016). A role of perception is to facilitate actions and interactions with the environment for humans or animals performing the activities. Perception is not merely a passive activity where one is made aware of a scenery, comparable to watching a movie at the cinema. Rather, perception has evolved as a powerful means to guide our activities and performances of those activities.

## Data Availability

The original contributions presented in the study are included in the article/supplementary material, further inquiries can be directed to the corresponding author.

## Footnotes

^1^Or which properties experience presents to us.

^2^Naney uses a different term than ‘affordance’ to distance himself from Gibson’s view.

^3^According to Gibson, affordances are more salient to us than standard low-level properties such as colors and shapes.

^4^There is also a related debate about whether rich content in perception involves cognitive penetration as some arguments for the rich-view seem to rely on the idea that non-perceptual mental states can penetrate the content of visual experiences (see Siegel, 2010, p. 10; Montague, 2017, 2023 for discussions). In contrast, the view that we can experience affordances needs not commit us to a particular view of cognitive penetration. While it seems that the rich-view commits us to, at least a mild version of intellectualism, this is not the case for experiencing affordances. Experiencing that the hole in the ground affords hiding and that the apple is edible need not require conceptual competence, and such experiences likely occur in animals and people (see Chemero, 2003; Wagman et al., 2016 for arguments for animals’ experiences of affordances).

^5^There are several reasons for accepting weak Russellian content: Two experiences can represent the world to be in the same way even if they are experiences of numerically different objects, and two experiences can represent the world in the same manner even if one of them is a hallucination in which no object is perceived (Tye, 1995; Tye, 2000).
